# Evaluation of trypan blue stain in a haemocytometer for rapid detection of cerebrospinal fluid sterility in HIV patients with cryptococcal meningitis

**DOI:** 10.1186/s12866-017-1093-4

**Published:** 2017-08-22

**Authors:** Richard Kwizera, Andrew Akampurira, Tadeo K. Kandole, Kirsten Nielsen, Andrew Kambugu, David B. Meya, David R. Boulware, Joshua Rhein, on behalf of the ASTRO-CM Study Team

**Affiliations:** 1 Infectious Diseases Institute, College of Health Sciences, Makerere University, P.O.BOX 22418 Kampala, Uganda; 2 University of Minnesota, Minneapolis, Minnesota USA; 3 Department of Medicine, School of Medicine, College of Health Sciences, Makerere University, Kampala, Uganda

**Keywords:** Trypan blue, Cryptococcal meningitis, HIV, Diagnostic techniques and procedures, Point-of-care systems

## Abstract

**Background:**

Quantitative culture is the most common method to determine the fungal burden and sterility of cerebrospinal fluid (CSF) among persons with cryptococcal meningitis. A major drawback of cultures is a long turnaround-time. Recent evidence demonstrates that live and dead *Cryptococcus* yeasts can be distinguished using trypan blue staining. We hypothesized that trypan blue staining combined with haemocytometer counting may provide a rapid estimation of quantitative culture count and detection of CSF sterility. To test this, we evaluated 194 CSF specimens from 96 HIV-infected participants with cryptococcal meningitis in Kampala, Uganda. Cryptococcal meningitis was diagnosed by CSF cryptococcal antigen (CRAG). We stained CSF with trypan blue and quantified yeasts using a haemocytometer. We compared the haemocytometer readings versus quantitative *Cryptococcus* CSF cultures.

**Results:**

Haemocytometer counting with trypan blue staining had a sensitivity of 98% (64/65), while CSF cultures had a sensitivity of 95% (62/65) with reference to CSF CRAG for diagnostic CSF specimens. For samples that were positive in both tests, the haemocytometer had higher readings compared to culture. For diagnostic specimens, the median of log_10_ transformed counts were 5.59 (*n* = 64, IQR = 5.09 to 6.05) for haemocytometer and 4.98 (*n* = 62, IQR = 3.75 to 5.79) for culture; while the overall median counts were 5.35 (*n* = 189, IQR = 4.78–5.84) for haemocytometer and 3.99 (*n* = 151, IQR = 2.59–5.14) for cultures. The percentage agreement with culture sterility was 2.4% (1/42). Counts among non-sterile follow-up specimens had a median of 5.38 (*n* = 86, IQR = 4.74 to 6.03) for haemocytometer and 2.89 (*n* = 89, IQR = 2.11 to 4.38) for culture. At diagnosis, CSF quantitative cultures correlated with haemocytometer counts (R^2^ = 0.59, *P* < 0.001). At 7–14 days, quantitative cultures did not correlate with haemocytometer counts (R^2^ = 0.43, *P* = 0.4).

**Conclusion:**

Despite a positive correlation, the haemocytometer counts with trypan blue staining did not predict the outcome of quantitative cultures in patients receiving antifungal therapy.

**Electronic supplementary material:**

The online version of this article (doi:10.1186/s12866-017-1093-4) contains supplementary material, which is available to authorized users.

## Background

Cryptococcal meningitis (CM) accounts for 15–25% of AIDS-related deaths in Africa, with the majority of cases known to occur in sub-Saharan Africa [1–4]. Culture of cerebrospinal fluid (CSF) is currently the gold standard for diagnosis and the only way of determining both sterility of CSF and viability of *Cryptococcus* yeasts in CSF [3, 5]. Yeast cells observed in CSF and stained using India ink may fail to grow in culture, likely reflecting non-viability of cells with successful treatment. Since *Cryptococcus* cultures have a long turnaround time of up to 10–14 days, rapid evaluation of treatment success or the diagnosis of relapse is often delayed.

Quantitative microscopy using trypan blue staining has been used extensively as a dye exclusion staining technique (live cells exclude the dye) to distinguish between live and dead mammalian cells [6]. Recent studies performed on experimental (spiked) samples indicate that trypan blue stain may be beneficial in distinguishing between viable and dead cryptococcal cells in persons with cryptococcal meningitis [7].

We aimed to determine a simple method of quantification of live cryptococcal cells during the first 14 days of treatment following diagnosis. We hypothesized that direct microscopy on CSF using trypan blue in a haemocytometer may provide a more rapid way of quantifying viable cryptococcal cells in CSF or at least predict the outcome of quantitative culture. We tested this hypothesis in a real-life scenario using fresh undiluted CSF from HIV patients being managed for cryptococcal meningitis in an active clinical setting.

## Methods

### Study population

The population for this study included patients diagnosed and managed with cryptococcal meningitis at Mulago Hospital in Kampala, Uganda during the Adjunctive Sertraline for the Treatment of HIV-Associated Cryptococcal Meningitis (ASTRO-CM) Clinical Trial (ClincalTrials.gov: NCT01802385) pilot phase [8]. All participants were HIV-infected, ≥18 years, with a positive CSF CRAG. Lumbar punctures were performed at days 0, 3, 7, 14 and as clinically indicated as part of standard care for the control of raised intracranial pressure.

### Ethics statement

All research participants or their surrogates provided written informed consent. Ethical approval occurred from the Uganda National Council of Science and Technology (UNCST), Mulago Hospital Research and Ethics Committee, and the University of Minnesota.

### Study procedures

Cryptococcal meningitis was diagnosed by CSF cryptococcal antigen (CRAG) lateral flow assay (Immy, Norman, Oklahoma, USA), and only patients with a positive CSF CRAG were considered for inclusion. CSF was collected at serial time points and as clinically indicated in inpatient symptomatic adults being managed for cryptococcal meningitis (Additional file 1). We stained whole CSF with 0.4% trypan blue and quantified viable cryptococcal cells with a haemocytometer to estimate the number of viable cryptococcal cells per milliliter (cells/ml). Quantitative fungal cultures were performed on whole CSF and plates incubated at 30 °C for 10 days on Sabouraud dextrose agar (SDA), as previously described [9]. We compared haemocytometer counts (cells/ml) to the number of CFU/ml in culture, with an assumption that one CFU originates from one viable cell.

### Preliminary validation tests

Prior to comparing the haemocytometer to culture, we prepared fungal media containing SDA, chloramphenicol and 0.4% trypan blue stain. We then cultured known cryptococcal isolates on this medium to check whether viable yeast cells would take up the trypan blue stain. For viability testing, we initially incubated known cryptococcal isolates at a 0.5 McFarland dilution for 24 h at 55 °C to kill the yeast cells. The heat-killed cells were then treated with 0.4% trypan blue stain. Similarly, we treated one cryptococcal isolate with amphotericin B for 24 h to kill the yeasts. The isolate treated with amphotericin B was then mixed with trypan blue and observed under a light microscope.

### Trypan blue staining

Whole CSF was thoroughly mixed and a 1:2 dilution was made with trypan blue stain (15 μl of whole CSF and 15 μl of 0.4% trypan blue stain) in a cryovial. The mixture was gently mixed and left to stand at room temperature for 5 min. 10 μl of the mixture was then applied to the edge of a haemocytometer counting chamber between the cover slip and chamber. The mixture was drawn into the chamber by capillary action and left to sit for 1–2 min before counting.

Cells were counted using a 40× objective and snap shot images were taken for different fields. With the assumption that dead cells take up the stain while viable cells do not take up the stain, only viable (unstained) cryptococcal cells were counted in each of the 4 corner quadrants. An average of these 4 readings was obtained and multiplied by 10^4^ to obtain the number of viable cells per ml in the sample and doubled to account for the 1:2 dilution.

### Statistical analysis

A linear regression was performed to compare the log_10_ transformed readings of trypan blue stain using a haemocytometer (live cells/ml) to log_10_ transformed quantitative fungal cultures (CFU/ml). Counts at diagnosis were compared to counts at other proceeding visits using a McNemar’s paired t-test statistic. Data was analyzed using STATA version 13 (STATA, College Station, Texas). Analysis was aimed at establishing the level of agreement and deviation of haemocytometer counts from quantitative cultures in the determination of CSF sterility at 95% confidence interval (CI).

## Results

### Characteristics of the study population

Between March 2015 and December 2015, we enrolled 96 participants with cryptococcal meningitis (diagnosed by CSF CRAG) who provided 194 CSF specimens (diagnostic and therapeutic lumbar punctures) in Kampala, Uganda. Only three participants had a negative CSF culture at screening but with a positive CSF CRAG. Of the 194 CRAG positive CSF samples, 33% (65/194) were obtained at diagnosis, with the remaining CSF samples obtained at day 3 (*n* = 22), day 7 (*n* = 9), day 14 (*n* = 10) and as clinically indicated (*n* = 88) during amphotericin therapy.

Demographics were similar to other cohorts of HIV-infected patients with cryptococcal meningitis and reflected the larger trial (Table 1) [8], with 38% (34/90) of the participants women and an overall median age of 35 years (IQR = 30–40 years). The median baseline CD4 T cell count was 14 cells/μL (IQR, 5–51; *n* = 92). Status of antiretroviral therapy (ART) reflected recent epidemiological trends, with 51% (49/96) of the participants having received antiretroviral therapy (ART) and 5.2% (5/96) having previously defaulted from ART at diagnosis. Among participants reporting a headache (*n* = 92), the median duration was 14 days (IQR = 7–30). The median CSF opening pressure at baseline was 322 mmH_2_O (*n* = 95, IQR = 210–440).

**Table 1:** Baseline characteristics of the study population at CM diagnosis

Baseline parameters at CM diagnosis	N	Statistic
Females^a^, n (%)	90	34 (38)
Age^a^ (years), median(IQR)	90	35 (30–40)
On ART at CM diagnosis, n (%)	96	49 (51)
Had Defaulted ART at CM diagnosis, n (%)	96	5 (5.2)
Baseline CD4^a^ (cells/mL), median(IQR)	92	14 (5–51)
Headache, n (%)	96	92 (95.8)
Headache duration^a^ (days), median (IQR)	92	14 (7–30)
CSF Opening pressures^a^ (mmH_2_O), median(IQR)	95	322 (210–440)
Weight^a^ (Kgs), median (IQR)	84	51 (48.5–60)
CSF WBC’s/mL^a^, median (IQR)	89	4 (4–45)
Wasting^a^, n (%)	87	43 (49.4)

Data presented are percentages (%), medians and interquartile ranges (IQR)

*CM* cryptococcal meningitis, *ART* antiretroviral therapy, *CSF* cerebrospinal fluid, *WBC* white blood cells, *N* number of participants with data for each parameter

^a^Some parameters have *N* < 96 due to missing data

### Preliminary validation results on trypan blue stain

**Fig. 1:**
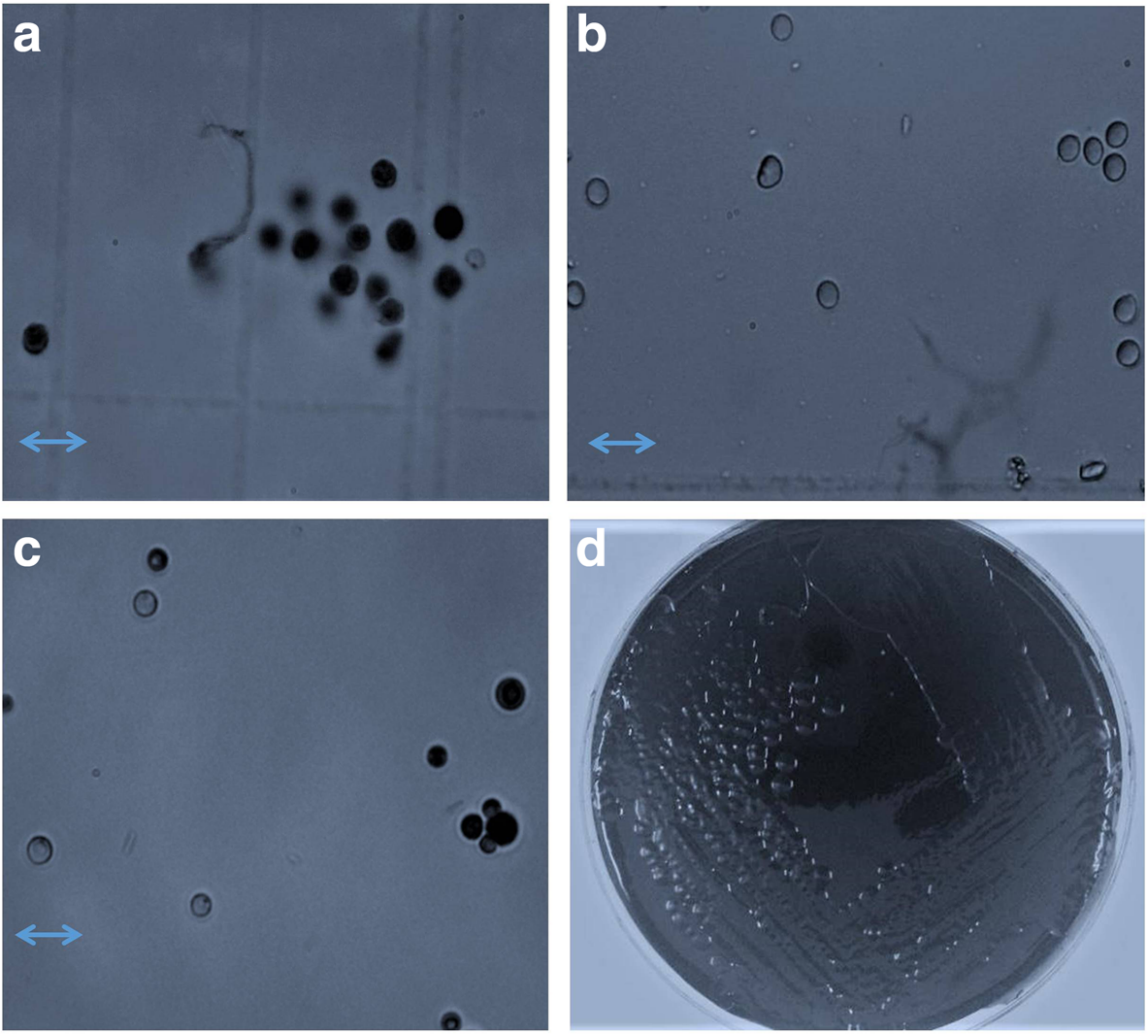
*Cryptoccocus* yeasts stained with Trypan blue. **a** Image of heat killed *Cryptococcus* yeasts stained with trypan blue and visualized by light microscopy. The dead cells appear dark/black. **b** Image of *Cryptococcus* yeasts left at room temperature and stained with trypan blue. The live cells appear light/white. **c** Image of a patient’s CSF sample stained with trypan blue. There is a mixed population of dead and live cells. **d** *Cryptococcus* isolate grown on media containing SDA, chloramphenicol and trypan blue. The colonies appear *dark blue*. “Scale bar = 10 μm”

To validate that trypan blue readily strains dead *Cryptococcus* cells, heat-killed *Cryptococcus* cells were stained with trypan blue and analysed by microscopy. All the heat-killed *Cryptococcus* cells took up the trypan blue stain (Fig. 1a), however cells in a similar preparation of live cells left at room temperature for 24 h did not take up the trypan blue stain (Fig. 1b). Patients’ samples showed mixed populations of live and dead cells (Fig. 1c). On the plates containing fungal medium (SDA), an antibiotic (chloramphenicol) and trypan blue stain, the *Cryptococcus* isolates grew dark blue colonies (Fig. 1d). However, when the dark blue colonies were viewed in a wet preparation under a light microscope, all the cells were viable, and none had taken up the trypan blue stain. The isolate treated with amphotericin B, when mixed with trypan blue and observed under a light microscope, showed that 98% of the cells were live and not killed by the antifungal treatment.

### Haemocytometer vs quantitative fungal cultures

Using CSF CRAG as a diagnostic reference, trypan blue staining with haemocytometer counting had a sensitivity of 98% (64/65) while CSF cultures had a sensitivity of 95% (62/65) for diagnostic CSF specimens. Among CSF samples collected longitudinally during antifungal treatment (all samples, including at diagnosis), the haemocytometer had more positive readings (97% [189/194]) than quantitative fungal culture (78% [151/194]) with reference to CSF CRAG. Among samples with no culture growth (*n* = 42), only one had a zero reading with the haemocytometer and the rest (*n* = 41) ranged in 5000 to 3,405,000 cells/ml. In other words, haemocytometer counting with trypan blue staining for viable *Cryptococcus* had excellent overall sensitivity of 97% but only agreed in 2% (1/42) of sterile cultures (McNemar’s *P* < 0.001). The positive predictive value for haemocytometer with reference to culture was 78% (147/189) while the negative predictive value (the probability that a zero reading by haemocytometer would have a negative culture) was only 20% (1/5).

When comparing the readings of trypan blue stain using a haemocytometer (log_10_-transformed live cells/ml) to quantitative fungal cultures (log10-transformed CFU/ml), culture consistently had lower counts than haemocytometer. The overall median counts were 5.35 log_10_ yeasts/mL (IQR = 4.78–5.84; max = 7.58; *n* = 189) for haemocytometer counting of trypan blue stained cells and 3.99 log_10_ CFU/mL (IQR = 2.59–5.14; max = 7.71; *n* = 151) for quantitative cultures. For diagnostic specimens only, the median of log10 transformed counts were 5.59 (*n* = 64, IQR = 5.09 to 6.05) for haemocytometer and 4.98 (*n* = 62, IQR = 3.75 to 5.79) for culture. Among 151 cultures with growth, there was a significant difference in the means of counts between culture and haemocytometer (*p* < 0.001); though there was a significant relationship between haemocytometer and culture overall by linear regression (R^2^ = 0.23, 95%CI: 0.16–0.30, *P* < 0.0001).

**Fig. 2:**
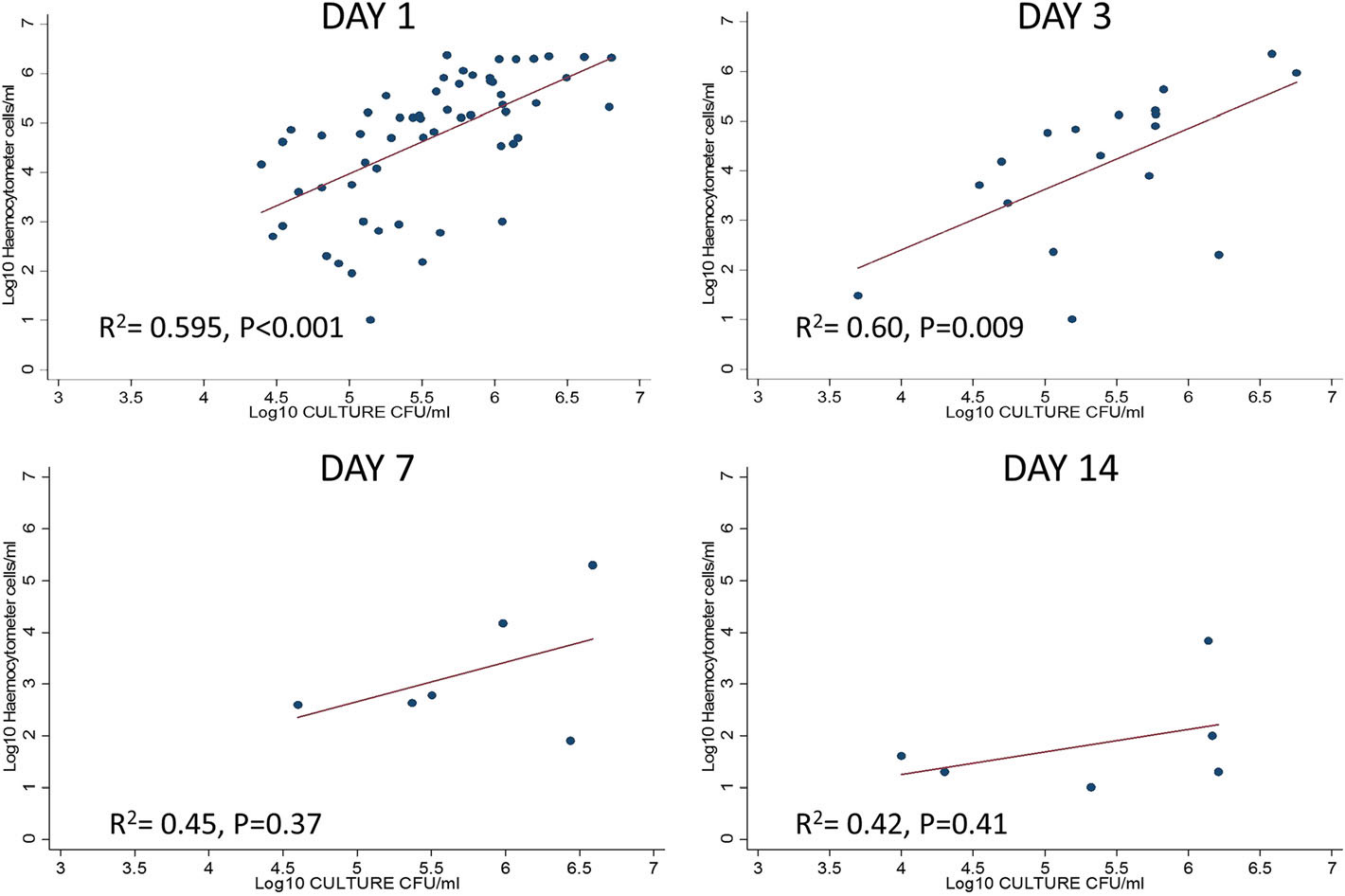
Haemocytometer counts vs quantitative culture. Scatter plots of haemocytometer manual cell counts vs quantitative culture from days 1, 3, 7 and 14 during antifungal treatment. A positive correlation was observed at days 1 and 3 (R^2^ = 0.595; 0.6 respectively) but was not significant at days 7 and 14 (R^2^ = 0.45; 0.42 respectively)

There was a significant correlation between the haemocytometer and culture readings at day 1 (R^2^ = 0.595, *P* < 0.001) and day 3 (R^2^ = 0.60, *P* = 0.009) (Fig. 2). However, this correlation did not extend through day 7 (R^2^ = 0.45, *P* = 0.37) or day 14 (R^2^ = 0.42, *P* = 0.41) of amphotericin B therapy. There was no significant relationship between the haemocytometer counts with trypan blue and baseline opening pressures (R2 = −0.17, *p* = 0.19). However, there was a significant correlation between culture and opening pressures (R^2^ = 0.34, *p* = 0.011).

## Discussion

Despite a significant relationship between the two methods and a positive correlation in the readings during the first week of treatment, the haemocytometer counts with trypan blue staining did not predict the outcome of quantitative culture, and culture had significantly lower values than the haemocytometer. However, linear regression showed a significant relationship (*p* < 0.0001, R^2^ = 0.60) between the two methods at day 1 and 3.

In our preliminary studies, we worked on the assumption that viable yeast cells with intact cell membranes would not take up the trypan blue stain while the stain can traverse the membrane of dead yeast cells. A recent publication from Australia showed similar results using *C. neoformans var. grubii* reference strain H99 [7]. In earlier work by *Vickers* et al. using fungal media with antibiotics and trypan blue to culture yeasts, *C. neoformans* showed dark blue colonies while *Candida albicans* showed light-white colonies. It was concluded that viable cells of *C. neoformans* take up the trypan stain [10]. However, a reproduction of this experiment in our laboratory revealed that all the yeast cells in the dark blue colonies were viable and none had taken up the stain in a wet preparation. It is possible that in both instances, the stain was associated with the external surface of the cell but not internalized into the cell.

When compared to cryptococcal cultures, the haemocytometer with trypan blue staining in most cases had higher values than culture. This could possibly be explained by “the Great Plate Count Anomaly” [11] since most fungi are known to be inherently fastidious in nature. Given the selectivity of the yeast cell membrane, live cells with intact cell membranes would not be expected to take up trypan blue whereas the stain would be expected to readily traverse the membrane of dead cells. This study included CSF cultures obtained in the context of patients with cryptococcal meningitis receiving active antifungal therapy. It is possible that dying yeast cells are too “unhealthy” or less viable to grow in culture yet have a cell membrane that remains intact enough to exclude trypan blue dye.

For both haemocytometer counts and culture, the fungal burden decreased over time while receiving amphotericin-based combination therapy. However, we observed significant differences (*P* = 0.001) between the two counting methods which became more pronounced with time on antifungal therapy. Furthermore, trypan blue staining was completely unreliable at predicting conversion to culture sterility. The clinical implication given these results is that staining with trypan blue cannot be used as a rapid surrogate for CSF cultures to help determine duration of therapy or early discontinuation of amphotericin in cryptococcal meningitis. Whether there could still be a role for trypan blue or other vital staining techniques to distinguish between culture-confirmed relapse and immune reconstitution inflammatory syndrome (IRIS) with sterile CSF is unknown. It is possible that trypan blue uptake could be more complete in yeast cells that have been dead for longer periods of time and have not yet been cleared from CSF, as has been observed in IRIS using traditional staining techniques. We tested this by treating one cryptococcal isolate with amphotericin B for 24 h to kill the yeasts with an antifungal agent. The isolate treated with amphotericin B, when mixed with trypan blue and observed under a light microscope, showed that 98% of the yeast cells were live and not killed by the antifungal treatment after 24 h.

A recent study done by Bio-Rad Laboratories using HeLa cells compared the haemocytometer to the TC20 automated counter. Results revealed that automated cell counting could significantly reduce both user and concentration-dependent variances while reducing the turn-around-time [12]. Whether automated cytometers could detect subtle differences in trypan blue staining and thus provide more accuracy remains unknown. Based on our observed differences between CSF trypan blue uptake and cell viability in culture, it is unlikely that automated counts would provide better prediction of sterility. With continued efforts to find an optimal method to rapidly detect CSF sterility among patients with cryptococcal meningitis, Acridine orange fluorescent microscopy showed better performance than India ink among CRAG positive HIV patients [13]. This study showed that fluorescent microscopy may greatly improve prediction of CSF sterility especially in sub-Saharan African where the disease remains a burden to the health systems.

## Conclusion

In conclusion, trypan blue staining using manual platforms was not helpful in the rapid detection of sterility of CSF and viability of *Cryptococcus* yeasts in the CSF.

## Additional file

Additional file 1: Study raw data. File contains a full set of raw data for the tests performed and baseline clinical and demographics characteristics. (XLSX 570 kb)

## Abbreviations

AIDS: Acquired immune deficiency syndrome
ART: Antiretroviral therapy
ASTRO-CM: Adjunctive Sertraline for the Treatment of HIV-Associated Cryptococcal Meningitis
*C.neoformans*: *Cryptococcus neoformans*
CD4: Cluster of differentiation 4
CFU/ml: Colony forming units per millilitre
CI: Confidence interval
CM: Cryptococcal meningitis
CRAG: Cryptococcal antigen
CSF: Cerebrospinal fluid
HIV: Human immunodeficiency virus
IQR: Interquartile range
IRIS: Immune reconstitution inflammatory syndrome
ml: Millilitre
n: Sample size
SDA: Sabouraud dextrose agar
UNCST: Uganda national council of science and technology
USA: United States of America
μl: Microliters
